# Association study of *IGFBP1* and *IGFBP3* polymorphisms with hypertension and cardio-cerebral vascular diseases in a Chinese Han population

**DOI:** 10.18632/oncotarget.20839

**Published:** 2017-09-12

**Authors:** Zhengmei Fang, Song Yang, Lijun Zhu, Ying Li, Yanchun Chen, Yuelong Jin, Xianghai Zhao, Hailong Zhao, Xiaotian Chen, Yanping Zhao, Chong Shen, Yingshui Yao

**Affiliations:** ^1^ Department of Epidemiology and Biostatistics, School of Public Health, Wannan Medical College, Wuhu 241001, China; ^2^ Department of Cardiology, Affiliated Yixing People's Hospital of Jiangsu University, People's Hospital of Yixing City, Yixing 214200, China; ^3^ Department of Epidemiology, School of Public Health, Nanjing Medical University, Nanjing 211166, China; ^4^ Central Laboratory, Affiliated Yixing People's Hospital of Jiangsu University, People's Hospital of Yixing City, Yixing 214200, China; ^5^ Department of Neurology, Affiliated Yixing People's Hospital of Jiangsu University, People's Hospital of Yixing City, Yixing 214200, China

**Keywords:** hypertension, IGFBP1, IGFBP3, cardio-cerebral vascular disease, polymorphisms

## Abstract

Previous studies have showed that insulin-like growth factor (IGF) axis is involved in the development of hypertension. It is unclear whether genetic variants in the IGF-binding proteins (IGFBPs) contribute to the susceptibility to hypertension. Three single-nucleotide polymorphisms (SNPs) in *IGFBP1* and four SNPs in *IGFBP3* were selected for genotyping in 2,012 hypertension cases and 2,210 healthy controls and 4,128 subjects were followed up for a median of 5.01 years. Multiple logistic regression and Cox regression were performed to evaluate the association of these seven SNPs with hypertension and cardio-cerebral vascular disease (CCVD). In the case-control study, rs2132572 and rs3110697 at *IGFBP3* were significantly associated with hypertension, and the odds ratios (ORs) of rs2132572 (CT+TT vs. CC) and rs3110697 (GA+AA vs. GG) were 1.235 (*P*=0.002) and 1.176 (*P*=0.013), respectively (*P*FDR<0.05). The association of rs2132572 (TT vs. CT+CC) with hypertension was further replicated in the follow-up population, with a hazard ratio (HR) of 1.694 (*P*=0.014). rs1874479 at *IGFBP1* was significantly associated with CCVD, particularly with stroke, and the HRs of the additive model were 1.310 (*P*=0.007) and 1.372 (*P*=0.015). Moreover, the hypertension cases presented with lower serum IGFBP1 levels than the controls (*P*=0.011). The serum levels of IGFBP1 significantly varied among the genotypes of rs1065780, rs2854843 and rs13223993, both in the controls and in the hypertension cases (*P*<0.05). These findings suggest that the genetic variants of *IGFBP1* and *IGFBP3* were associated with an increased risk of stroke and hypertension, respectively. Lower serum IGFBP1 levels may predict an increased risk of hypertension.

## INTRODUCTION

Hypertension is a global public health challenge due to its high prevalence. The total number of hypertensive cases in adults is predicted to reach 1.5 billion by 2025 [1]. The severe outcome of hypertension is primarily due to its driving role in the development of cardio-cerebral vascular disease (CCVD), including coronary heart disease (CHD) and stroke [2]. Hypertension is a complex and multi-causal trait, and both genetic and environmental determinants contribute to its pathogenesis [3].

Insulin-like growth factor-1 (IGF-1) promotes the somatic growth, proliferation and migration of vascular smooth muscle cells (VSMC), and cellular survival [4]. IGF-binding proteins (IGFBP1~6) inhibit IGF-1 interactions with their receptors and prolong the half-lives of IGF-1 [5]. IGFBP1 expression was significantly up-regulated in induced acute hypotension rats [6]. Animal experiment suggested that IGFBP1 stimulates nitric oxide production by activating the PI3K/Akt/phospho-eNOS pathway and subsequently reducing blood pressure [7].

Elevated IGFBP1 levels are linked to higher CHD risk [8] and increased cardiovascular disease (CVD) mortality [9, 10]. Lower IGFBP1 levels are associated with hypertension [11] and CVD risk factors [12]. A higher IGFBP3 level was associated with hypertension [13, 14], CHD [15], ischemic heart disease [16], and atherosclerosis [17]. In contrast, other studies reported an association between lower IGFBP3 levels and stroke [18], coronary events [19] and CVD mortality [20]. These inconsistent results suggest that the association of IGFBPs with CCVD needs to be further elucidated.

Genetic variants of *IGFBP3* were found to be associated with circulating levels of IGFBP3 [21–24]. A genome-wide association study (GWAS) found that rs11977526 near the *IGFBP1/IGFBP3* was associated with higher IGFBP3 levels and lower IGF1 levels [25]. A twin study indicated that 60% of the IGFBP3 variance was attributable to the genetic effects of *IGFBP3* [26]. A GWAS meta-analyses showed that *IGFBP3* near the single-nucleotide polymorphism (SNP) rs2949837, was significantly associated with increased long-term average pulse blood pressures in a population with European ancestry [27]. Furthermore, rs11977526 was associated with decreased systolic blood pressure (SBP), increased diastolic blood pressure (DBP) and hypertension in an African ancestry population [28]. The association of rs11977526 and hypertension was further replicated in an east African population [29]. Moreover, rs2854744 polymorphisms in *IGFBP3* were associated with a decreased risk of stroke in the patients with coronary artery disease of the Chinese population [30]. Therefore, *IGFBP1* and *IGFBP3* are of particular interest as candidate genes for hypertension.

Agnieszka et al [33] reported that in a small sample of perimenopausal women with hypertension (n=152) and normotensive controls (n=40), IGFBP2 levels were inversely correlated with hypertension and metabolic syndrome. Whereas the association of IGFBP2 level with hypertension were not adjusted for lipid levels. Thus further replication is warranted. IGFBP4, IGFBP5 and IGFBP6 have yet not been researched for human hypertension. Herein, we evaluated the association of seven SNPs of *IGFBP1* and *IGFBP3* with hypertension in a community-based case-control study of the Chinese Han population as well as with CCVD in a prospective follow-up study.

## RESULTS

### Demographic characteristics

A total of 2,012 hypertensive cases and 2,210 normotensives adults were included in this case-control study. The demographic and clinical characteristics of participants are shown in Table 1. The gender, HDL, smoking status, and drinking status between two groups were not significantly different. The hypertension cases had higher mean SBP, DBP, TC, TG, LDL-C, GLU, and BMI than the controls (P<0.001). The hypertension cases were slightly older (by 3.42 years) than the controls (P<0.001), although an age-matched (5 years) method was used for analysis.

**Table 1:** Comparison of the demographic and clinical characteristics of the hypertension cases and controls

Characteristics	Group	Case-control study			*P*
		Normotensive (n=2210)	Hypertension (n=2012)	*t/χ^2^*	
Gender	Male	884 (40%)	829 (41.2%)	0.632	0.427
	Female	1326 (60%)	1183 (58.8%)		
Age (year)		58.93±10.45	62.35±10.73	10.484	<0.001
Blood press (mmHg)	SBP	124.24±11.36	142.86±14.3	46.523	<0.001
	DBP	79.08±6.51	87.53±8.54	35.918	<0.001
TC (mmol/L)		4.79±1.01	4.99±1.05	4.574	<0.001
TG (mmol/L)		1.54±1.21	1.87±1.58	7.526	<0.001
HDL-C (mmol/L)		1.36±0.33	1.37±0.33	0.175	0.861
LDL-C (mmol/L)		2.65±0.73	2.8±0.89	6.17	<0.001
GLU (mmol/L)		5.46±1.61	5.83±2.05	6.609	<0.001
BMI (kg/m^2^)		23.64±3.2	24.76±3.51	10.798	<0.001
Smoking	Yes	533 (24.1%)	480 (23.9%)	0.039	0.843
	No	1677 (75.9%)	1532 (76.1%)		
Drinking	Yes	476 (21.5%)	423 (21%)		
	No	1734 (78.5%)	1589 (79.0%)	0.166	0.683

During a median follow-up period of 5.01 years, 613, 183, 106, 268, and 86 individuals developed hypertension, stroke, CHD, CCVD, and CCVM, respectively, with an incidence density of 656.84, 86.19, 50.85, 126.52, and 39.89 per 10^4^ person-years.

### Association analyses of *IGFBP1* and *IGFBP3* polymorphisms with hypertension

In the case-control study, the genotype distributions of seven tagSNPs were in accordance with HWE (*P*>0.05) in the control population. rs2132572 and rs3110697 of *IGFBP3* showed significant associations with hypertension in the whole study population (Supplementary Table1). After adjustment for age, gender, TC, TG, LDL-C, HDL-C, GLU, BMI, smoking status, and drinking status, the adjusted OR (95%CI) of rs2132572 (CT +TT vs. CC) was 1.235 (1.081-1.411), *P*=0.002 and *P*_FDR_=0.014. The adjusted OR (95%CI) of rs3110697 (GA +AA vs. GG) was 1.176 (1.035-1.336), *P*=0.013 and *P*_FDR_=0.046 (Table 2).

**Table 2:** Association analyses of *IGFBP* and hypertension in the case-control study

SNP	Group	WT/HT/MT	Genotypes OR (95%CI)*			*P*-HWE
			Additive	Dominant	Recessive	
rs1065780	Control	661/1091/455	0.974(0.89-1.065)	0.984(0.857-1.129)	0.942(0.806-1.102)	0.901
(A>G)	Case	607/1001/404	*P*=0.562	*P*=0.814	*P*=0.457	
rs2854843	Control	964/991/254	1.058(0.963-1.163)	1.105(0.972-1.255)	1.007(0.827-1.226)	0.977
(T>C)	Case	829/949/234	*P*=0.243	*P*=0.126	*P*=0.942	
rs1874479	Control	1461/666/80	1.057(0.944-1.184)	1.063(0.931-1.214)	1.094(0.782-1.53)	0.704
(A>G)	Case	1290/646/76	*P*=0.339	*P*=0.363	*P*=0.599	
rs3110697	Control	1299/771/133	1.099(0.99-1.22)	1.176(1.035-1.336)	0.908(0.691-1.192)	0.194
(G>A)	Case	1115/785/110	*P*=0.076	*P*=0.013	*P*=0.486	
rs13223993	Control	939/1007/259	1.052(0.957-1.156)	1.093(0.962-1.243)	1.011(0.832-1.228)	0.66
(G>A)	Case	810/960/240	*P*=0.291	*P*=0.173	*P*=0.915	
rs2132572	Control	1505/622/77	1.167(1.041-1.309)	1.235(1.081-1.411)	0.992(0.703-1.399)	0.202
(C>T)	Case	1283/654/72	*P*=0.008	*P*=0.002	*P*=0.962	
rs2453839	Control	1363/738/105	1.105(0.993-1.23)	1.117(0.982-1.271)	1.192(0.895-1.587)	0.691
(T>C)	Case	1196/703/112	*P*=0.066	*P*=0.093	*P*=0.23	

WT, wild type; HT, heterozygote; MT, mutant type; FDR, false discovery rate.

*Adjusted for age, gender, TC, TG, HDL-C, LDL-C, GLU, BMI, drinking and smoking.

In the follow-up study of hypertension, the association of rs2132572 with hypertension was further replicated (Supplementary Table 2). After adjustment for age, gender, TC, TG, LDL-C, HDL-C, diabetes, BMI, smoking status, and drinking status, the adjusted HR (95%CI) of rs2132572 (TT vs. CT + CC) was 1.694 (1.113-2.579), *P*=0.014 (Table 3). No significant association was observed between the variants at *IGFBP1* and the incidence of hypertension (Supplementary Tables 1 and 2).

**Table 3:** Association analyses of *IGFBP* and hypertension incidence in the follow-up study

SNP	Genotypes	Case (n)	ID (per 10^5^person-years)	Genotypes HR (95%CI)*		
				Additive	Dominant	Recessive
rs1065780	AA	181	6535.12	1.029(0.918-1.154)	1.056(0.886-1.259)	1.017(0.832-1.242)
	AG	309	6681.11	*P*=0.622	*P*=0.543	*P*=0.869
	GG	122	6321.77			
rs2854843	TT	279	6752.91	0.894(0.792-1.008)	0.887(0.755-1.043)	0.812(0.625-1.055)
	TC	270	6525.14	*P*=0.068	*P*=0.147	*P*=0.118
	CC	64	6037.28			
rs1874479	AA	425	6788.05	0.935(0.803-1.09)	0.946(0.795-1.125)	0.768(0.451-1.308)
	AG	173	6324.58	*P*=0.391	*P*=0.528	*P*=0.332
	GG	14	4237.8			
rs3110697	GG	355	6421.54	1.125(0.982-1.29)	1.092(0.928-1.285)	1.489(1.057-2.098)
	GA	219	6653.3	*P*=0.09	*P*=0.292	*P*=0.023
	AA	36	7388.71			
rs13223993	GG	261	6507.87	0.921(0.816-1.039)	0.926(0.787-1.09)	0.84(0.65-1.085)
	GA	284	6734.13	*P*=0.18	*P*=0.354	*P*=0.181
	AA	67	6129.36			
rs2132572	CC	415	6531.63	1.096(0.942-1.274)	1.047(0.881-1.244)	1.694(1.113-2.579)
	CT	173	6462.24	*P*=0.235	*P*=0.601	*P*=0.014
	TT	23	8107.73			
rs2453839	TT	379	4830.62	1.004(0.867-1.162)	0.985(0.834-1.162)	1.163(0.75-1.803)
	TC	211	6698.58	*P*=0.96	*P*=0.856	*P*=0.499
	CC	21	5363.44			

*Adjusted for age, gender, TC, TG, HDL-C, LDL-C, diabetes, BMI, drinking and smoking.

ID incidence density.

### Association analyses of *IGFBP1* and *IGFBP3* polymorphisms with CCVD incidence

**Figure 1:**
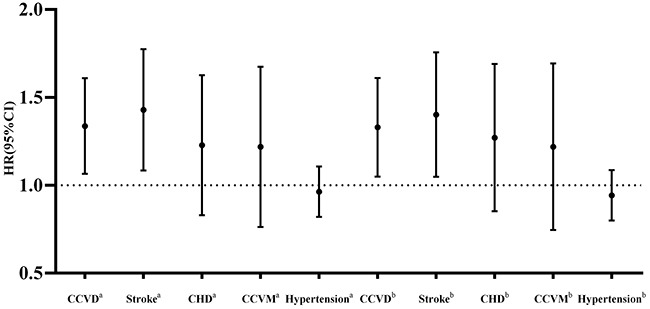
The HRs (95% CIs) of the additive model of rs1874479 for the risk of CCVD incidence, stroke, CHD, CCVM and hypertension in the follow-up study ^a^ Crude HRs (95% CIs); ^b^ HRs (95% CIs) with adjustments for age, gender, TC, TG, LDL-C, HDL-C, diabetes, BMI, smoking status, drinking status and hypertension (except for the incident hypertension).

The rs1874479 variation showed a significant association with CCVD incidence (Figure 1). The adjusted HR of the additive model was 1.310 (*P*=0.007), after adjustment for age, gender, TC, TG, LDL-C, HDL-C, diabetes, BMI, smoking status, drinking status, and hypertension. Particularly, the association of rs1874479 with stroke was significant, and the adjusted HR of the additive model was 1.372 (*P*=0.015). No significant association was observed between the other SNPs at *IGFBP1* and *IGFBP3* and incidence of CCVD or stroke (Supplementary Table 3).

### Comparison of serum IGFBP1 and IGFBP3 between hypertension cases and controls

The median (interquartile range) of serum IGFBP1 in the hypertension cases [16.77 (6.97, 50.35) ng/ml] was significantly lower than that in the controls [32.37 (10.12, 73.72) ng/ml], *P*=0.011. No significant difference in the serum IGFBP3 concentration was observed between the hypertension cases and the controls (*P*=0.112).

### Comparison of serum IGFBP1 and IGFBP3 among the genotypes

**Figure 2:**
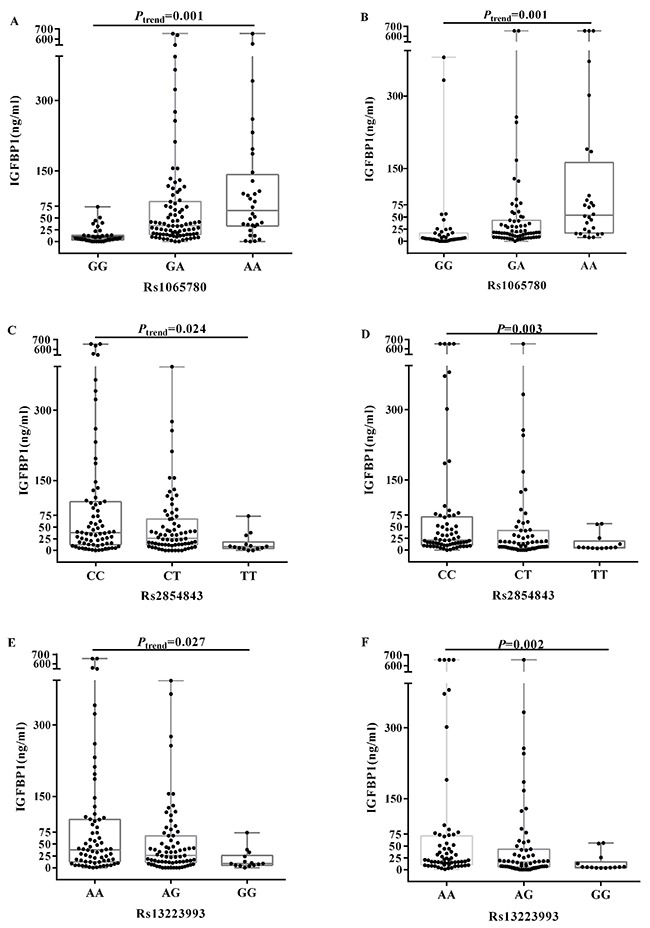
Serum IGFBP1 levels were compared among the genotypes of rs1065780, rs2854843 and rs13223993 in the controls and hypertension cases IGFBP1 levels are plotted around the median as box plots, and the dots represent individual data points. The diamonds and the whiskers represent the mean and SD of the IGFBP1 levels, respectively. Figure 2 **(A)**, **(C)** and **(E)** show the IGFBP1 levels among the genotypes in the controls, and **(B)**, **(D)** and **(F)** display the IGFBP1 levels among genotypes in the hypertension cases. The serum IGFBP1 levels linearly increased with the variations in rs1065780 both in the controls and the hypertension cases (*P*_trend_=0.001). They linearly decreased with variations of in rs2854843 and rs13223993 in the controls (*P*_trend_ <0.05). rs2854843 and rs13223993 variations were associated with lower IGFBP1 levels in the hypertension cases (all *P* <0.05).

Figure 2 summarizes the distribution of serum IGFBP1 levels among the genotypes of rs1065780, rs2854843 and rs13223993. Our results showed that the serum IGFBP1 levels linearly increased with the variations in rs1065780 both in the controls and in the hypertension cases (*P*_trend_=0.001). The serum IGFBP1 levels linearly decreased with the variations in rs2854843 and rs13223993 in the controls (*P*_trend_<0.05). rs2854843 and rs13223993 variations were associated with lower IGFBP1 levels in the hypertension cases (all *P* <0.05). All data are shown in Supplementary Table 4. No significant differences in the serum IGFBP3 levels were observed among the genotypes of seven SNPs (data are not shown).

## DISCUSSION

In the present study, we first showed that rs2132572 and rs3110697 at *IGFBP3* are associated with an increased risk of hypertension. The association between rs2132572 and hypertension was replicated for a median of 5.01 years of follow-up. The G allele of rs1874479 at *IGFBP1* was associated with an increased risk of incident stroke and CCVD, particularly stroke. Additionally, decreased IGFBP1 levels were observed in hypertension cases. With population-based evidence, these findings further support the role of IGFBPs in the development of cardiovascular diseases.

Previous studies have indicated that rs3110697 and rs2132572 polymorphisms are associated with decreased circulating levels of IGFBP3 [23, 24], but this association was not replicated in the current study. The IGFBP3 levels were not different between the hypertension cases and controls. Studies observed that both the variations of rs2132572 and rs3110697 were associated with increased IGF1 levels in the premenopausal women [31], and the variation in rs3110697 was associated with higher IGF1 levels in benign breast disease [32]. These results suggest that the risk effects of rs2132572 and rs3110697 on hypertension might be involved in influencing IGF1 concentrations rather than directly regulating IGFBP3 expression itself. These findings may help to unravel the complex genetics underlying disease predisposition and individual responses to therapeutics targeting the IGF-1 signaling pathway.

In the present analysis, several common variants of *IGFBP1* and *IGFBP3*, especially rs2854843 (intron) and rs1065780, presented strong associations with the serum IGFBP1. rs1065780, located in the promoter region of *IGFBP1*, is predicted to bind multiple transcription factors such as Nkx2.8 ( http://jaspar.genereg.net//), thus playing an important role in liver development [33]. The AA genotype of rs13223993 at *IGFBP3* presented lower mean IGFBP1 levels. rs13223993, located at the 3’ UTR of *IGFBP3*, has been shown to regulate protein expression through miRNAs or proteins, and stabilize or destabilize the mRNA half-life [34]. Given their tail-to-tail fashion location [35], it might be worth investigating the cooperation mechanism of the two genes in the IGF1 pathway.

Additionally, the variation of rs1874479 at IGFBP1 presented an increased risk of CCVD incidence. In particular, rs1874479 showed a significant association with the incidence of stroke, but not with CHD. In Hawaiian women, mammography density analysis showed that women with at least 1 copy of the minor allele for rs1874479 had 5.7 percent greater breast density [36], but no biological function was mentioned. Further functional research is therefore needed to characterize this polymorphism.

This study has several limitations. First, the lack of significance between IGFBP3 SNPs and serum IGFBP3 could be due in part to the smaller sample size with reduced power. Second, the three SNPs associated with lower IGFBP1 in hypertension did not show a direct association with the hypertension risk. Finally, the relationship between IGFBP polymorphisms and CCVD needs further investigation for longer follow-up periods due to the relatively low CCVD incidence.

In summary, the findings of this study suggest that hypertensive cases have lower serum IGFBP1 levels than the controls. *IGFBP1* and *IGFBP3* genetic variants are associated with varied IGFBP1 level. rs2132572 and rs3110697 in *IGFBP3* presented statistical associations with an increased risk of hypertension. rs1874479 in *IGFBP1* was associated with a risk of incident CCVD, particularly stroke. Further functional research is needed to validate these findings.

## MATERIALS AND METHODS

### Subjects

The case-control study included 2,012 hypertensive cases and 2,210 normotensive participants recruited from May to October 2009, in the Jiangsu province [37] and the subjects were followed up from April 2014 to September 2016. Hypertension was defined as SBP ≥140 mmHg and/or DBP ≥90 mmHg, as well as the self-reported diagnosis of hypertension, or the current usage of antihypertensive medications.

**Figure 3:**
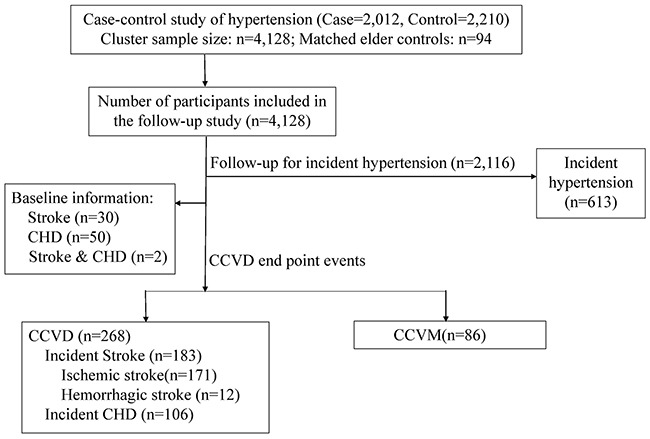
2012 hypertension cases and 2210 healthy controls were assessed. 4,128 subjects were further followed up, for a median of 5.01 years

In the follow-up study, 94 elderly matched controls for the case-control study were excluded. A total of 4128 subjects participated in the follow-up study, and a total of 2116 healthy controls were included. Participants were prospectively followed during a median follow-up period of 5.01 years to assess the incidence of hypertension, CCVD and CCVD mortality (CCVM) events, including stroke and CHD (Figure 3).

The study procedures were approved by the ethics committee of Nanjing Medical University, and all participants provided written informed consent during the epidemiological interviews.

### Epidemiological interviews and anthropometric measurements

Trained research staff administered a standard questionnaire to obtain the demographic characteristics of the participants, including their age, gender, nationality, education level, smoking and drinking habits, and medical history. All participants received physical examinations, and the body mass index (BMI; weight (kg)/height (m)^2^) and BP of the participants were measured. Three BP measurement readings were obtained for each participant by trained and certified observers according to a standard protocol [37]. Participants were asked about their smoking and drinking habits. Smoking was defined having at least 20 cigarettes per week for 3 months per year. Drinking was defined as drinking at least 2 times per week for 6 months per year. Peripheral venous blood was drawn from participants after 10 hours of overnight fasting to measure the high-density lipoprotein cholesterol (HDL-C), low-density lipoprotein cholesterol (LDL-C), total cholesterol (TC), triglycerides (TG), and glucose (GLU).

### IGFBP1 and IGFBP3 detection

In a randomized sample including 136 incident hypertension cases that did not receive any antihypertensive treatment and 157 healthy controls, the serum IGFBP1 was measured using a human IGFBP1 enzyme-linked immunosorbent assay (ELISA) KIT (Catalog NO: CSB-EO4586h, CASABIO, China). Serum IGFBP3 was measured using the human IGFBP3 ELISA KIT (Catalog NO: CSB-EO4590h, CASABIO, China).

### SNPs selection

*IGFBP1* maps to chromosome 7q12.3 (Gene ID: 3484; Locus NC_000007.14) and spans 6.9 kbps with 4 exons. *IGFBP3* is located on chromosome 7q12.3 (Gene ID: 3486; Locus NC_000007.14), spans 12 kbps and consists of 5 exons. We searched through the database of the Han Chinese population in Beijing, and the human reference genome (GRCh37, http://gvs.gs.washington.edu/GVS147/). We applied the linkage disequilibrium (LD) method to select tagged SNPs (tagSNPs) with r^2^≥0.8 as candidate SNPs. Three SNPs (rs1065780, rs2854843, rs1874479) at *IGFBP1* and four SNPs (rs3110697, rs2132572, rs13223993, rs2453839) at *IGFBP3* were selected with minor allele frequencies (MAF) ≥0.05. Furthermore, the SNP functional consequence was predicted using the National Institute of Environmental Health Sciences (NIEHS) SNPinfo website (SNPINFO, https://snpinfo.niehs.nih.gov/) and the JASPAR database ( http://jaspar.genereg.net//).

### Genotyping

DNA was extracted using a standard phenol–chloroform method. DNA concentration and the purity of each sample were measured using the Thermo Scientific NanoDrop 2000 spectrophotometer. Amplification of all seven SNPs in *IGFBP1* and *IGFBP3* were performed using a polymerase chain reaction (PCR)-TaqMan MGB probe array in the GeneAmp® PCR system 9700 (Applied Biosystems, USA) thermal cycler with dual 384-well-blocks according to the manufacturer's instructions. PCR reactions were performed in a 5μl reaction mixture that included 10ng DNA and 2.4μl 2×TaqMan® Universal PCR Master Mix. The endpoint plates were read on the ABI 7900 system using the Sequence Detection System (SDS) 2.1 software (Applied BioSystems, Foster City, CA). The successful call rates of SNPs were over 99.8%.

### Statistical analysis

Measurement variables were presented as the means ± standard difference (SD) between the cases and controls, and a t-test was used to test their differences. Categorical variables between the cases and controls were compared by the Chi square (χ^2^) test. Fisher's exact χ^2^test using the program Hardy–Weinberg equilibrium (HWE) was performed to estimate the HWE in the control group. Multiple logistic regression analysis was applied to calculate the odds ratio (OR) and its 95% interval confidence (CI) and adjust for covariates. The hazard ratio (HR) of associations in the follow-up study was estimated by Cox proportional hazard regression. IGFBP1 and IGFBP3 did not follow Gaussian distributions, so a nonparametric test was used to assess the differences between hypertension cases and controls. Statistical analyses were performed using the SPSS version 18.0 (SPSS, Inc, Chicago, IL). A two-tailed P value of 0.05 was defined to be statistically significant. The false discovery rate (FDR) was estimated by using the Benjamini-Hochberg procedure to correct the P-values for multiple comparisons.

## SUPPLEMENTARY MATERIALS FIGURES AND TABLES




