# Supplementary material for: Association study of IGFBP1 and IGFBP3 polymorphisms with hypertension and cardio-cerebral vascular diseases in a Chinese Han population
Source: Oncotarget. 2017 Sep 12;8(44):77836–45. doi: 10.18632/oncotarget.20839 (PMC5652818; doi:10.18632/oncotarget.20839)
Supplement: Supplementary file 1 [file oncotarget-08-77836-s001.pdf]

Association study of *IGFBP1* and *IGFBP3* polymorphisms with hypertension and cardio-cerebral vascular diseases in a Chinese Han population

SUPPLEMENTARY MATERIALS

Supplementary Table 1: Association analyses of *IGFBPs* and hypertension in the case-control study

SNP	Group	WT/HT/MT	ORs (95%CIs)		
			Additive	Dominant	Recessive
rs1065780	control	661/1091/455	0.985(0.904-1.073)	0.99(0.867-1.129)	0.967(0.833-1.124)
(A>G)	case	607/1001/404	P=0.727	P=0.877	P=0.665
rs2854843	control	964/991/254	1.059(0.968-1.16)	1.105(0.978-1.249)	1.013(0.839-1.224)
(T>C)	case	829/949/234	P=0.211	P=0.11	P=0.894
rs1874479	control	1461/666/80	1.075(0.964-1.198)	1.096(0.966-1.244)	1.044(0.758-1.437)
(A>G)	case	1290/646/76	P=0.193	P=0.156	P=0.793
rs3110697	control	1299/771/133	1.083(0.98-1.197)	1.153(1.021-1.303)	0.901(0.695-1.169)
(G>A)	case	1115/785/110	P=0.116	P=0.022	P=0.433
rs13223993	control	939/1007/259	1.057(0.966-1.158)	1.099(0.972-1.242)	1.019(0.845-1.228)
(G>A)	case	810/960/240	P=0.228	P=0.132	P=0.845
rs2132572	control	1505/622/77	1.16(1.039-1.294)	1.218(1.072-1.384)	1.027(0.74-1.424)
(C>T)	case	1283/654/72	P=0.008	P=0.002	P=0.874
rs2453839	control	1363/738/105	1.093(0.987-1.211)	1.102(0.974-1.247)	1.18(0.898-1.551)
(T>C)	case	1196/703/112	P=0.087	P=0.125	P=0.235

WT, wild type; HT, heterozygote; MT, mutant type.

Supplementary Table 2: Association analyses of *IGFBPs* and hypertension incidence in the follow-up study

SNP	Genotypes	Incident case (n)	HRs (95%CIs)		
			Additive	Dominant	Recessive
rs1065780	AA	181	1.024(0.916-1.145)	1.088(0.914-1.295)	0.965(0.791-1.177)
	AG	309	p=0.676	P=0.344	P=0.726
	GG	122			
rs2854843	TT	279	0.919(0.816-1.034)	0.928(0.792-1.088)	0.817(0.63-1.06)
	TC	270	P=0.159	P=0.359	P=0.128
	CC	64			
rs1874479	AA	425	0.956(0.824-1.11)	0.992(0.835-1.179)	0.663(0.39-1.127)
	AG	173	P=0.557	P=0.93	P=0.129
	GG	14			
rs3110697	GG	355	1.118(0.979-1.278)	1.105(0.941-1.299)	1.346(0.961-1.885)
	GA	219	P=0.1	P=0.223	P=0.084
	AA	36			
rs13223993	GG	261	0.945(0.84-1.064)	0.968(0.824-1.136)	0.843(0.654-1.088)
	GA	284	P=0.349	P=0.688	P=0.189
	AA	67			
rs2132572	CC	415	1.108(0.956-1.285)	1.074(0.906-1.273)	1.57(1.034-2.383)
	CT	173	P=0.174	P=0.414	P=0.034
	TT	23			
rs2453839	TT	379	0.99(0.857-1.144)	0.974(0.827-1.148)	1.105(0.714-1.709)
	TC	211	P=0.892	P=0.754	P=0.655
	CC	21			

Supplementary Table 3: Association analyses of *IGFBPs* and CCVD, stroke, CHD and CCVM incidence in the follow-up study.

See Supplementary File 1

Supplementary Table 4: Distribution of IGFBP1 levels among the variants of rs1065780, rs2854843 and rs13223993genotypes

SNP		Normotensives		Hypertensives	
		N	median (IQR)	N	median (IQR)
rs1065780	AA	37	6.86(3.31,13.20)	42	5.31(3.38,17)
	AG	87	33.68(15.16,84.66)	67	18.11(8.65,43.21)
	GG	32	65.97(32.97,142.44)	28	53.80(16.90,162.54)
	P		<0.001		<0.001
	P_{trend}		0.001		0.001
rs2854843	TT	72	37.89(12.45,104.42)	66	20.94(10.4,70.97)
	TC	72	26.51(12.1,67.51)	58	15.74(4.83,42.23)
	CC	14	8.04(4.13,17.89)	13	5.45(4.43,19.32)
	P		0.002		0.003
	P_{trend}		0.024		0.089
rs13223993	GG	68	37.89(12.45,101.75)	61	21.12(10.3,71.73)
	GA	73	25.88(11.05,67.41)	59	16.46(5.96,43.22)
	AA	14	8.88(5.14,26.04)	14	5.31(4.12,16.11)
	P		0.008		0.002
	P_{trend}		0.027		0.071

IQR, Interquartile range.
