# Supplementary material for: Association study of IGFBP1 and IGFBP3 polymorphisms with hypertension and cardio-cerebral vascular diseases in a Chinese Han population
Source: Oncotarget. 2017 Sep 12;8(44):77836–45. doi: 10.18632/oncotarget.20839 (PMC5652818; doi:10.18632/oncotarget.20839)
Supplement: Supplementary file 2 [file oncotarget-08-77836-s002.docx]

| **Supplementary Table 3: Association analyses of *IGFBPs* and CCVD, stroke, CHD and CCVM incidence in the follow-up study** | | | | | | | | | | | | | | | | | |
| --- | --- | --- | --- | --- | --- | --- | --- | --- | --- | --- | --- | --- | --- | --- | --- | --- | --- |
| SNP |  | CCVD | | |  | Stroke | | |  | CHD | | |  | CCVM | | | |
|  |  | N | HRs(95%CI)^a^ | HRs(95%CI)^b^ |  | N | HRs(95%CI)^a^ | HRs(95%CI)^b^ |  | N | HRs(95%CI)^a^ | HRs(95%CI)^b^ |  | N | HRs(95%CI)^a^ | | HRs(95%CI)^b^ |
| rs1065780 | AA | 80 | 0.985(0.829-1.171) | 0.971(0.814-1.157) |  | 57 | 1.003(0.815-1.233) | 0.987(0.798-1.222) |  | 34 | 0.958(0.73-1.258) | 0.969(0.737-1.275) |  | 30 | 0.958(0.703-1.306) | 0.917(0.665-1.265) | |
|  | AG | 130 | *P*=0.868 | *P*=0.74 |  | 85 | *P*=0.934 | *P*=0.908 |  | 52 | *P*=0.758 | *P*=0.823 |  | 37 | *P*=0.788 | *P=*0.599 | |
|  | GG | 54 |  |  |  | 41 |  |  |  | 20 |  |  |  | 19 |  |  | |
| rs2854843 | TT | 109 | 1.031(0.861-1.234) | 1.007(0.84-1.208) |  | 74 | 1.067(0.86-1.322) | 1.049(0.842-1.307) |  | 44 | 1.025(0.769-1.366) | 0.99(0.743-1.319) |  | 41 | 0.821(0.59-1.144) | 0.815(0.579-1.145) | |
|  | TC | 120 | *P*=0.742 | *P*=0.938 |  | 83 | *P*=0.557 | *P*=0.67 |  | 49 | *P*=0.866 | *P*=0.944 |  | 35 | *P*=0.244 | *P=*0.238 | |
|  | CC | 35 |  |  |  | 26 |  |  |  | 13 |  |  |  | 10 |  |  | |
| rs1874479 | AA | 159 | 1.318(1.075-1.618) | 1.310(1.060-1.620) |  | 107 | 1.401(1.099-1.787) | 1.372(1.064-1.770) |  | 66 | 1.185(0.853-1.646) | 1.225(0.877-1.711) |  | 56 | 1.162(0.794-1.7) | 1.158(0.779-1.721) | |
|  | AG | 88 | *P*=0.008 | *P*=0.013 |  | 65 | *P*=0.007 | *P*=0.015 |  | 33 | *P*=0.313 | *P*=0.233 |  | 26 | *P*=0.441 | *P=*0.47 | |
|  | GG | 17 |  |  |  | 11 |  |  |  | 7 |  |  |  | 4 |  |  | |
| rs3110697 | GG | 147 | 1.029(0.838-1.264) | 1.026(0.836-1.26) |  | 97 | 1.099(0.864-1.397) | 1.125(0.884-1.433) |  | 62 | 0.924(0.664-1.288) | 0.911(0.653-1.27) |  | 47 | 0.984(0.674-1.435) | 0.981(0.672-1.431) | |
|  | GA | 104 | *P*=0.783 | *P*=0.804 |  | 76 | *P*=0.332 | *P*=0.339 |  | 40 | *P*=0.642 | *P*=0.582 |  | 36 | *P*=0.931 | *P=*0.921 | |
|  | AA | 13 |  |  |  | 10 |  |  |  | 4 |  |  |  | 3 |  |  | |
| rs13223993 | GG | 107 | 1.04(0.869-1.244) | 1.02(0.85-1.223) |  | 74 | 1.041(0.839-1.291) | 1.026(0.822-1.279) |  | 42 | 1.103(0.831-1.463) | 1.072(0.806-1.425) |  | 39 | 0.848(0.61-1.177) | 0.838(0.597-1.178) | |
|  | GA | 120 | *P*=0.669 | *P*=0.834 |  | 83 | *P*=0.716 | *P*=0.822 |  | 48 | *P*=0.498 | *P*=0.633 |  | 37 | *P*=0.324 | *P*=0.31 | |
|  | AA | 37 |  |  |  | 26 |  |  |  | 16 |  |  |  | 10 |  |  | |
| rs2132572 | CC | 168 | 1.053(0.846-1.312) | 1.053(0.846-1.310) |  | 113 | 1.106(0.852-1.434) | 1.107(0.855-1.433) |  | 68 | 1.063(0.752-1.502) | 1.045(0.739-1.479) |  | 55 | 1.002(0.673-1.493) | 1(0.671-1.489) | |
|  | CT | 84 | *P*=0.643 | *P*=0.646 |  | 61 | *P*=0.449 | *P*=0.440 |  | 34 | *P*=0.731 | *P*=0.802 |  | 27 | *P*=0.991 | *P=*0.998 | |
|  | TT | 11 |  |  |  | 8 |  |  |  | 4 |  |  |  | 4 |  |  | |
| rs2453839 | TT | 157 | 1.011(0.821-1.245) | 1.050(0.855-1.29) |  | 106 | 1.072(0.837-1.372) | 1.129(0.884-1.441) |  | 65 | 0.913(0.652-1.279) | 0.931(0.665-1.303) |  | 51 | 0.951(0.651-1.391) | 0.982(0.674-1.432) | |
|  | TC | 93 | *P*=0.918 | *P*=0.641 |  | 67 | *P*=0.583 | *P*=0.331 |  | 36 | *P*=0.597 | *P*=0.676 |  | 31 | *P*=0.797 | *P=*0.926 | |
|  | CC | 13 |  |  |  | 10 |  |  |  | 4 |  |  |  | 4 |  |  | |
| ^a^ Crude HR, ^b^ model was adjusted for age, gender, TC, TG, HDL-C, LDL-C, hypertension, diabetes, BMI, drinking and smoking, | | | | | | | | | | | | | | | | | |
